# Assessing the Use of Ustekinumab in a Pediatric Patient With Harlequin Ichthyosis

**DOI:** 10.7759/cureus.37654

**Published:** 2023-04-16

**Authors:** Nouf Almuhanna, Bushra S Alasmari, Rasha Alhamazani, Sarah Alkhezzi, Faris A Alhomida

**Affiliations:** 1 Dermatology, King Fahad Medical City, Riyadh, SAU

**Keywords:** genetic skin disorders, genetic syndromes, autoimmune and genetic skin diseases, retinoid, pediatric dermatology, treatment choices, biologic agents, biologic treatment, congenital ichthyosis, harlequin ichthyosis

## Abstract

Harlequin ichthyosis (HI) is a rare, life-threatening genodermatosis that is characterized by thick, scaly, hyperkeratotic plaques throughout the skin and is typically associated with severe ectropion, eclabium, flexion contractures, and dysplastic ears. HI is thought to be caused by a loss-of-function mutation in the ABCA12 gene. It has traditionally been thought to be difficult to treat, as there are currently no treatments available that are approved by the Food and Drug Administration (FDA). We present a case of a 15-year-old boy with HI and a complex medical history who was treated with a trial of off-label ustekinumab. There was an initial mild improvement in his erythema within one month of treatment, but by his one-year follow-up, ustekinumab had failed to produce a significant treatment response and was, thus, discontinued from his regimen. This case report highlights that although ustekinumab may be a viable treatment option for other ichthyotic entities, more research is needed to evaluate its clinical safety and efficacy in treating pediatric patients with HI.

## Introduction

Harlequin ichthyosis (HI) is a rare, life-threatening genodermatosis that affects approximately one in every 300,000 live births. It is typically characterized by thick, scaly, hyperkeratotic plaques and tends to be classically associated with severe ectropion, eclabium, flexion contractures, and dysplastic ears. HI is considered one of the severe forms of autosomal recessive congenital ichthyosis. A loss-of-function mutation in the ABCA12 gene, which encodes an essential protein for lipid transport in the formation of the skin's protective barrier, is responsible for this condition [1,2].

Neonates with HI have a reported 50% mortality rate, primarily due to a compromised skin barrier, resulting in dehydration, electrolyte imbalances, thermodysregulation, and a high risk of sepsis. In addition, the presence of thick hyperkeratotic plaques may limit chest expansion, thereby potentially increasing the risk of respiratory failure and contributing to the high mortality rate [1,3].

HI has traditionally been thought to be difficult to treat, as there are no treatments currently available that are approved by the Food and Drug Administration (FDA).

## Case presentation

A 15-year-old boy with HI was referred to our dermatology clinic for further management of his condition. Genetic testing was done at an outside facility that revealed a homozygous mutation in the ABCA12 gene. Thus, confirming the diagnosis of HI. He had a complicated past medical history and was born small for gestational age at full term to consanguineous parents. He had a prolonged one-year stay at the neonatal intensive care unit due to his condition. His past medical history is notable for developmental delay, short stature, failure to thrive requiring gastrostomy tube feeds, flexion contractures, osteopenia, left corneal ulceration, and bilateral cicatricial lagophthalmos. His past surgical history is notable for laparoscopic orchiopexy, ectropion repair, and tonsillectomy. His family history is also notable for diabetes and hypertension in his maternal grandmother. He has no other relevant family history and no other affected family members. His medication list includes somatropin 1.5 mg once daily by subcutaneous injection (34 mcg/kg/day), 25 mg daily of oral acitretin, topical nightly tazarotene 0.05%, topical as needed 40% urea cream, topical corticosteroids, 10-20 mg oral as needed hydroxyzine at bedtime, topical emollients, and topical artificial tears. He has no known drug allergies.

**Figure 1:**
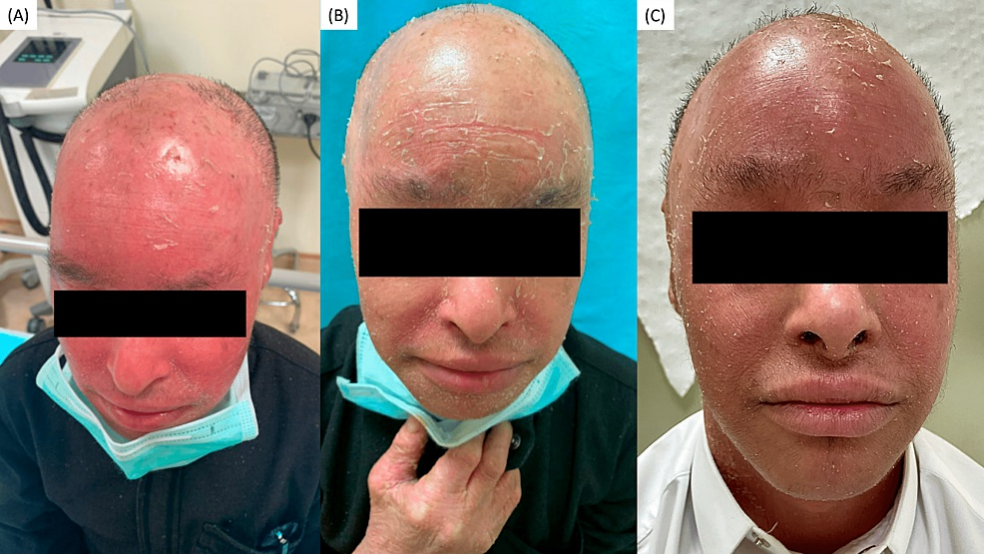
The patients state at baseline, one month, and one year after starting ustekinumab A: baseline before ustekinumab; scattered yellow-brown hyperkeratotic plaques with desquamation on a background of diffuse erythema on the face and scalp. B: one month after the first dose of ustekinumab, note the reduction of erythema but an increase in the hyperkeratotic plaques. C: one year after starting ustekinumab, although arguably less erythematous, his condition is essentially unchanged from baseline.

At his presentation in our dermatology clinic, he stands at 1.47 m (<3^rd^ percentile) with a weight of 40.9 kg (<3^rd^ percentile) and is vitally stable [4]. His cutaneous examination was notable for scattered, yellow-brown, scaly, hyperkeratotic plaques with desquamation on a background of diffuse erythema throughout his skin (Figure 1A).

Mild bilateral ectropion, bilateral flexion contractions of the thumb, and bilateral dysplastic ears were also noted. There was no eclabium noted on the exam. He had an affected body surface area of more than 90%, with an Ichthyosis Scoring System (ISS) score of 4.017 and a Peak Pruritus Numerical Rating Scale (NRS) score of 5 [5,6]. 

Laboratory work-up (Table 1) revealed a normal complete blood count, renal function test, hepatic function test, lipid panel, hepatitis B and C screening, and QuantiFERON®-TB Gold testing (ARUP Laboratories, Salt Lake City, US).

**Table 1:** Laboratory analysis revealed a normal work-up

Test	Component	Reference range and units	Results
Complete blood count	White blood cell	>3.90 - <11.00 10*3/uL	4.01
	Red blood cell	>4.50 - <6.00 10*6/uL	5
	Hemoglobin	13.5 - 18 g/dL	13.7
	Hematocrit	>37 - <52%	46
	Mean corpuscular volume	>75.0 - <95.0 fL	79.6
	Mean corpuscular hemoglobin	>24.0 - <30.0 pg	27.4
	Red blood cell distribution width	>11 - <15.0 %	13.6
	Mean platelet volume	>6.3 - <11.2 fL	9
	Platelets	100 - 450 10*3/uL	208
Renal function test	Sodium	136.00 - 145.00 mmol/L	136
	Potassium	3.50 - 4.50 mmol/L	3.76
	Calcium	1.63 - 3.53 mmol/L	2.43
	Chloride	98.00 - 107.00 mmol/L	104
	Bicarbonate	20.00 - 28.00 mmol/L	21.96
	Albumin	32.00 - 45.00 g/L	41.4
	Urea	3.00 - 7.50 mmol/L	5.8
	Creatinine	26. 00 - 62.00 umol/L	56
Hepatic function test	Alkaline phosphatase	89.00 - 365.00 U/L	196
	Alanine transaminase	0.00 - 55.00 U/L	14
	Aspartate aminotransferase	5.00 - 34.00 U/L	19
	Total bilirubin	1.71 - 20.5 µmol/L	6.9
Lipid panel	Triglycerides	<1.70 mmol/L	1.03
	Cholesterol	<4.400 mmol/L	3.49
	High-density lipoprotein	>1.55 mmol/L	1.73
	Low-density lipoprotein	<2.60 mmol/L	2.27
Hepatitis B screening	Hepatitis B surface antigen	Negative	Negative
	Hepatitis B surface antibody	<10.00 mIU/ml	9.97
	Anti-hepatitis B core antibody	Negative	Negative
Hepatitis C screening	Hepatitis C antibody	Negative	Negative
Tuberculosis screening	QuantiFERON-TB Gold	Negative	Negative

Given his lack of improvement with his current regimen, we elected to start him on a trial of off-label, ustekinumab 45 mg subcutaneous injections at weeks zero, four, then every 12 weeks. Despite medical advice, his father elected to withhold acitretin therapy while starting ustekinumab. The patient initially reported a decrease in erythema after his first dose of ustekinumab (Figure 1B), but there was no change in his NRS score of 5 or ISS score of 4.017, most likely as a result of an increase in the hyperkeratosis following the discontinuation of acitretin. Due to the lack of improvement, acitretin was then re-added to his regimen. By one-year follow-up, due to a lack of response, ustekinumab was discontinued from his regimen. He reported a worsening of his pruritus with an NRS score of 7, and a negligible 1.64% decrease was noted in his ISS score of 3.951 (Figure 1C).

He continues on his current regimen of systemic and topical retinoids, topical keratolytics, topical corticosteroids, emollients, and systemic antihistamines. Furthermore, he continues to follow up with pediatric dermatology, ophthalmology, endocrinology, and the nutrition team.

## Discussion

Treatment of HI in the pediatric population is challenging, given its rarity and the lack of treatments approved by the FDA. Treatments are usually based on symptomatic management with the goal of controlling hyperkeratosis. Treatment regimens typically consist of topical bland emollients, such as petroleum-based products, and keratolytics, like alpha-hydroxy acids, urea, or topical retinoids for the hyperkeratotic plaques. However, some patients may not be able to tolerate the stinging or irritation associated with keratolytics. In addition, the early use of systemic retinoids has been reported to be life-saving. Systemic retinoids have been shown to not only help promote the shedding of hyperkeratotic plaques but may also improve both the digital and thoracic constrictions, leading to improved breathing and functional movement. Additionally, systemic retinoids have also been shown to decrease the risk of digital necrosis [1,3].

Promising developments may be on the horizon with corrective gene therapy as a possible treatment option for patients with HI. However, clinical trials need to be done to assess the efficacy and safety of such measures. Furthermore, recent findings regarding the immune profile of ichthyosis patients have opened up new avenues for repurposing the use of biologics. Biologics such as secukinumab, ustekinumab, and dupilumab have all shown promising results in treating other ichthyotic entities, namely, Netherton syndrome and Lamellar ichthyosis [7]. Furthermore, Yogarajah et al. have previously reported the successful use of secukinumab in a pediatric patient with a heterozygous ABCA12 deficiency-related ichthyosis. They reported a 48% reduction from the baseline Ichthyosis Area Severity Index score within six months of treatment with secukinumab [8].

Ustekinumab is a fully human, monoclonal IgG1 antibody that selectively inhibits interleukin-12 and interleukin-23 by binding to the shared p40 subunit. It has been FDA-approved for the treatment of plaque psoriasis, psoriatic arthritis, Crohn's disease, and ulcerative colitis [9]. Clinical trials are currently underway to examine the use of ustekinumab in patients with various Ichthyoses. More time is needed, however, before the results become available [10].

To our knowledge, this is the first reported use of ustekinumab in a pediatric patient with HI. Although our patient showed an initial improvement in his erythema, it remains unclear if this improvement is attributed to the use of ustekinumab or the discontinuation of his acitretin. By one-year follow-up, however, despite resuming acitretin, there was no treatment response, and his pruritus worsened. This ultimately led to ustekinumab being discontinued from his regimen.

Hence, additional research measures are imperatively needed to thoroughly examine the safety and efficacy of ustekinumab prior to endorsing its utilization as a viable treatment option for patients with HI.

## Conclusions

The management of HI in pediatric patients remains a significant challenge due to its rarity and the absence of FDA-approved treatments. This case report highlights the first use of ustekinumab in a pediatric patient with HI, which showed an initial improvement in erythema but ultimately failed to demonstrate a sustained treatment response. Although biological therapy may offer a promising avenue for future treatment options, further research is essential to establish their safety and efficacy in treating HI in pediatric patients. In the meantime, clinicians must continue to rely on symptomatic management with a multidisciplinary approach to best optimize patient outcomes.
